# Research on Optimizing the Steel Fiber/CSH Interface Performance Based on Ca/Si Ratio

**DOI:** 10.3390/ma18174049

**Published:** 2025-08-29

**Authors:** Yalin Luan, Yongmei Wu, Runan Wang, Dongbo Cai, Lianzhen Zhang, Pengxiang Luan

**Affiliations:** 1 The Seventh Engineering Co., Ltd. of CCCC First Highway Engineering Co., Ltd., Zhengzhou 451452, China; luanyalin@upc.edu.cn (Y.L.); 19854212857@163.com (Y.W.); 15583130944@163.com (D.C.); 2 College of Pipeline and Civil Engineering, China University of Petroleum (East China), Qingdao 266580, China; 19862857721@163.com; 3 Wuxi National Hi-Tech Development District SCI-TECH Innovation Promotion Center, Wuxi 214142, China; luanpx@foxmail.com

**Keywords:** steel fiber reinforced concrete, calcium-to-silicon ratio, γ–FeOOH/CSH nanopore, molecular dynamics simulations

## Abstract

Steel fiber reinforced concrete in marine environments often suffers from stress corrosion coupling. Under mechanical loading, the formation of penetrating cracks in the matrix increases susceptibility to seawater penetration and interfacial degradation. Using molecular dynamics simulations, this study investigated the effects of calcium-to-silicon (Ca/Si) ratios on the interfacial bonding and transport properties of a γ-FeOOH/CSH system. The results show that higher Ca/Si ratios strengthen ionic bonding between CSH and γ-FeOOH, thereby improving interfacial adhesion. Additionally, increased Ca/Si ratios significantly slow the transport of water molecules and ions (Na^+^, Cl^−^, SO_4_^2−^) within γ-FeOOH/CSH nanopores. It was observed that Cl^−^ and SO_4_^2−^ exhibited pronounced filtration effects at Ca/Si = 2.0. These findings suggest that optimizing the Ca/Si ratio in concrete can simultaneously enhance interfacial strength and reduce permeability. This provides an effective strategy for improving the marine erosion resistance of steel fiber reinforced concrete structures.

## 1. Introduction

As an essential building material, concrete plays a crucial role in marine engineering construction. The durability of concrete structures is a critical factor in determining the service life of port infrastructure [1,2,3]. The incorporation of steel fibers into concrete enhances its mechanical properties and durability, making steel fiber reinforced concrete widely used in marine environments [4,5,6,7]. However, micro-gaps at the steel fiber/cement matrix interface create a mechanically weak zone that promotes fiber debonding and crack bridging, enhancing toughness [8,9]. When seawater penetrates the interface, chloride ions (Cl^−^) play a critical role by depassivating steel fibers and inducing corrosion. However, coexisting ions such as Na^+^ and SO_4_^2−^ may further exacerbate degradation through synergistic effects; Na^+^ increases ionic conductivity, while SO_4_^2−^ reacts with cement hydrates, collectively reducing interfacial adhesion and impermeability [10,11,12]. This degradation leads to a progressive decline in the macroscopic performance of concrete, ultimately compromising the long-term safety of marine concrete structures [13,14,15,16,17]. While experimental methods are limited by scale constraints, molecular dynamics (MD) simulations can bridge the gap between macroscopic experiments and microscopic mechanisms, providing critical insights for improving concrete durability at the fundamental level [18,19].

Current research has focused on modification of concrete interfacial properties to address its inherent weaknesses. Some studies have explored material composition adjustments: Hou et al. [20] investigated the interaction of water molecules with calcium silicate hydrate (CSH) gels at varying calcium-to-silicon (Ca/Si) ratios. The results indicate that low-Ca/Si CSH has a denser structure, which results in larger water contact angles due to hindered penetration into silicate chains. Thus, higher Ca/Si ratios improve wettability. Wu et al. [21] conducted uniaxial tensile tests on four CSH gel models with Ca/Si ratios (1.24, 1.41, 1.64 and 1.80), revealing that increased Ca/Si reduced the Young’s modulus and peak stress of CSH, weakening tensile performance. Zhou et al. [22] combined experiments and simulations to demonstrate that higher Ca/Si ratios break long silicate chains into shorter fragments, creating more non-bridging oxygen sites that promote water and Ca^2+^ accumulation, thereby enhancing chloride adsorption.

Other studies have employed interfacial adhesives to improve performance. Yang Qingrui et al. [23] showed that graphene nanosheets can form a “idge” the epoxy/CSH interface, enhancing the interfacial bonding performance through Ca_CSH_–O_GO_–H_epoxy_, O_CSH_–H_GO_–O_epoxy_ (N_epoxy_), and H_CSH_–O_GO_–H_epoxy_ bonds. Guo et al. [24] found that graphene oxide slit size affects CSH formation; larger slits facilitate Si and Ca^2+^ adsorption and promote polymerization, while smaller slits cause structural inhomogeneity. Yang et al. [25] pointed out that the addition of 3–(aminopropyl) triethoxysilane (3–APTES) could change the fracture process of CSH, increase the atomic density, and enhance its tensile toughness and adsorption capacity for water molecules.

The studies mentioned above provide two strategies for optimizing the steel fiber/CSH interface. On one hand, the molecular structure of CSH can be changed by adjusting the material ratio, such as constructing CSH models with different Ca/Si. On the other hand, molecular bond connections can be strengthened by incorporating interface enhancers such as graphene oxides and 3–APTES. However, existing work primarily focuses on the mechanical, wetting, and transport properties of CSH matrix, with insufficient attention to steel fiber/CSH interfacial adhesion and ion transport. Furthermore, there is a lack of comprehensive simulations on steel fiber concrete with varying compositions.

To address these gaps, this study employed MD simulations to investigate the steel fiber/CSH interface in a NaCl + Na_2_SO_4_ composite solution environment. Four CSH crystal models with Ca/Si ratios of 1.3, 1.5, 1.7, and 2.0 were constructed to form γ–FeOOH/CSH interfacial bonding–transport systems. The interfacial adhesion was evaluated through interaction energy analysis and water/ion binding mechanisms, while transport properties were assessed via ion penetration depth and local bonding effects. Finally, suggestions for optimizing the performance of steel fiber/CSH interface are proposed from the perspective of Ca/Si ratio selection. The research aims to determine how different Ca/Si ratios affect the interfacial bonding strength and ion transport behavior in the γ–FeOOH/CSH system, providing insights for optimizing concrete formulations to enhance corrosion resistance in marine environments.

## 2. Materials and Methods

### 2.1. Model Construction

Tobermorite 11 Å crystal served as the initial structure for modeling CSH with varying Ca/Si ratios. To achieve different ratios, the Q_n_ species distribution in the silicate chains was adjusted based on NMR data [26], and electrically neutral SiO_2_ groups were removed to shorten the silicate chains [27]. Increasing the Ca/Si ratio introduced more micropores between the truncated chains. Water adsorption was then simulated using Grand Canonical Monte Carlo (GCMC) at 300 K with a chemical potential of 0 eV, employing the CSH–FF force field [28].

The system reached saturation after 100 million simulation steps. Each CSH model was sliced along the (0 0 1) interlayer direction, and surface water was removed to minimize interference. Surface Ca^2+^ and hydroxyl groups were adjusted to maintain charge balance, with consistent Ca^2+^ content across models to preserve the target Ca/Si ratios. lower Ca/Si ratios resulted in longer silicate chains and higher Q^2^_b_ abundance (where Q represents Si–O tetrahedron, 2 indicates the number of other Q units to which it is bonded increases, b represents the bridging Si). The Q species distribution for each model is summarized in Table 1 [20].

**Figure 1:**
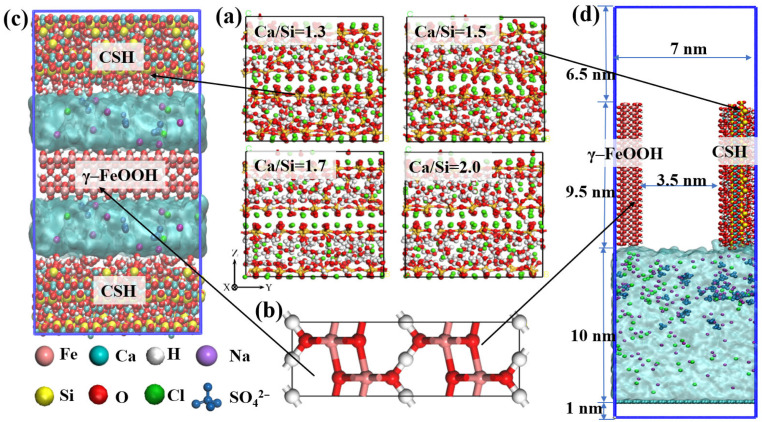
(**a**) Different Ca/Si CSH models, (**b**) γ–FeOOH, (**c**) γ–FeOOH/CSH interface bonding model and the blue square represents the boundary of the box (**d**) γ–FeOOH/CSH interface transport model.

The steel fiber/CSH interface models with different Ca/Si ratios were constructed using three primary components. Firstly, four distinct tobermorite crystal structures with Ca/Si ratios of 1.3, 1.5, 1.7, and 2.0 were established, as shown in Figure 1a. These CSH structures were modified to represent realistic hydration products through the selective removal of H_2_O molecules and random elimination of bridging Si–O tetrahedra within the silicate chains [28,29]. Secondly, the steel fiber component was modeled using γ–FeOOH, which represents the primary constituent of the passive film formed on steel surfaces under the strong alkaline conditions [30] created by calcium hydroxide in cement hydration products [31]. This approach accounts for long-term environmental effects on steel fiber surfaces [32,33], as illustrated in Figure 1b. Thirdly, an aqueous solution environment was created using a NaCl + Na_2_SO_4_ mixture with a density of 1.04 g/cm^3^, to simulate realistic marine conditions [34].

The four CSH structures and γ–FeOOH structure were expanded along their crystallographic axes to form CSH/γ–FeOOH/CSH sandwich configurations. A 12.5 Å vacuum layer was maintained between the γ–FeOOH and CSH surfaces [35], which was subsequently filled with the NaCl + Na_2_SO_4_ composite solution to establish the interfacial bonding model in Figure 1c. In the transport model, the expanded sizes of CSH and γ–FeOOH structures with four Ca/Si ratios were 22.6 Å × 95 Å × 20 Å and 22.6 Å × 95 Å × 15 Å, respectively [36]. CSH and γ–FeOOH structures were positioned on either side of a central 3.5 nm–wide channel, with the erosive solution placed at the channel outlet in Figure 1d.

### 2.2. Force Field and Molecular Dynamics Procedure

Molecular dynamics (MD) simulations were performed using the Large–scale Atomic Molecular Massively Parallel Simulator (LAMMPS) [37,38] free software package, with visualization of the processes realized by Visual Molecular Dynamics (VMD). The ClayFF force field [39] was used due to its general applicability for simulating various cement hydration products, interfaces between minerals and solutions, anion and cation adsorption on hydroxide surfaces, and the interatomic potentials between solution species and the calcium silicate skeleton.

The bonding simulation process was conducted as follows: a 300 ps NPT ensemble for all atoms was established for energy minimization. Then, a 1000 ps NVT ensemble simulation was applied to achieve complete system equilibration. The transmission simulation involved three stages, as follows. First, both CSH and γ–FeOOH walls were set as rigid bodies, i.e., in a “frozen” state [40], and an invisible wall was placed between the channel entrance and the solution to prevent water molecules and ions from escaping. Second, the solution was simulated for 200 ps in the NVT ensemble to achieve equilibrium [41]. Third, the “frozen” walls were released, and the invisible wall at the pore channel entrance was removed. A 2000 ps simulation was performed in the NVT ensemble, allowing water and ions to freely enter the pore. Periodic boundary conditions were applied in all simulations to eliminate size effects, and the temperature was maintained at 300 K using a Nosé–Hoover thermostat. Additionally, atomic coordinates were calculated every 0.1 ps, and the resulting atomic trajectories were used for structural and dynamic analysis.

## 3. Results and Discussion

### 3.1. Interaction Energy of γ–FeOOH/CSH Interface

**Figure 2:**
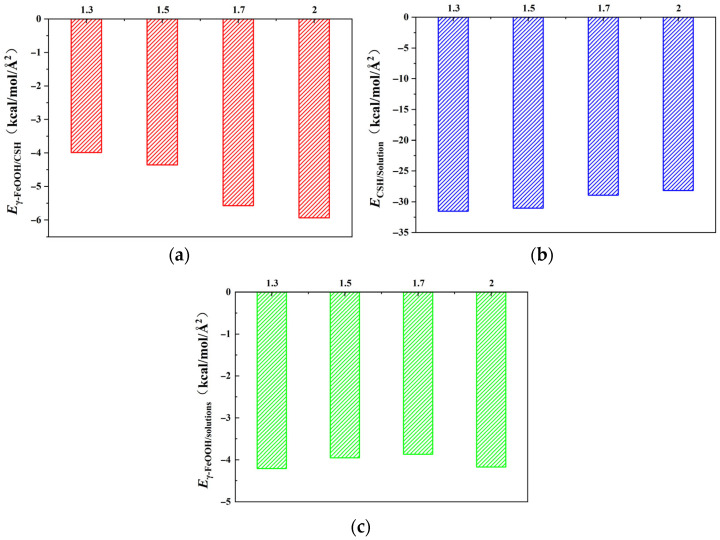
Interaction energy per unit contact area between different components. (**a**) γ–FeOOH and CSH; (**b**) CSH and solutions; (**c**) γ–FeOOH and solutions.

Interaction energy refers to the energy generated between molecules or atoms due to the interaction of various force fields (such as van der Waals forces, Coulomb forces, hydrogen bonds, etc.), and it is an important physical quantity that describes the strength of the interaction between molecules. To investigate interfacial adhesion properties of γ–FeOOH/CSH systems with varying Ca/Si ratios of 1.3, 1.5, 1.7, and 2.0, the unit area interaction energies between different components were calculated, using the following equation. The computational results are presented and analyzed below.

(1) $$E_{1/2}=\frac{E_{total}-(E_{1}+E_{2})}{A_{1/2}}$$

where *E*_1/2_ denotes the interaction energy per unit area between components 1 and 2; *E_total_* is the total energy of the system containing both components; *E*_1_ and *E*_2_ are the individual energies of components 1 and 2, respectively, and *A*_1/2_ represents the cross-sectional area between components 1 and 2. The calculated interaction energies are shown in Figure 2.

With the change of Ca/Si ratios, the interaction energies between each pair of components (γ–FeOOH/CSH, CSH/NaCl + Na_2_SO_4_ composite solution, and γ–FeOOH/solution) all exhibited a certain variation pattern. *E*_γ–FeOOH/CSH_ exhibited a gradual increase with increasing Ca/Si ratioe. When Ca/Si = 1.3, *E*_γ–FeOOH/CSH_ reached its minimum value of −3.987 kcal/mol·Å^2^. The negative value indicates mutual attraction between the two components. *E*_γ–FeOOH/CSH_ increased by 9.22%, 39.8%, and 48.7%, respectively, with increasing Ca/Si ratios. When Ca/Si = 2.0, *E*_γ–FeOOH/CSH_ reached a maximum of *E*_CSH/solutions_ = −5.937 kcal/mol/Å^2^. Conversely, *E*_CSH/solutions_ showed an opposite trend, with the maximum value of *E*_CSH/solutions_ = −31.537 kcal/mol·Å^2^ observed at Ca/Si = 1.3. As the Ca/Si ratio increased, *E*_CSH/solutions_ decreased by 1.56%, 8.15%, and 10.63%, respectively. *E*_γ–FeOOH/solutions_ remained relatively stable across different Ca/Si ratios, with values of −4.211, −3.952, −3.867, and −4.174 kcal/mol·Å^2^, indicating that structural variations in CSH had negligible effects on the interaction between γ–FeOOH and the corrosive solution.

The results above suggest that lower Ca/Si ratios in CSH promote stronger interactions with the NaCl + Na_2_SO_4_ composite solution, leading to increased adsorption of solution molecules on the CSH surface. This adsorption forms a solution barrier that hinders direct bonding between CSH and γ–FeOOH, consequently weakening their interfacial interaction [42,43]. In contrast, higher Ca/Si ratios facilitate stronger direct bonding between CSH and γ–FeOOH by reducing solution adsorption effects.

From the perspective of energy, the stronger attraction between CSH and γ–FeOOH in Figure 2a promotes direct bonding at higher Ca/Si ratios, creating a denser interfacial zone that physically obstructs the penetration of water and ions. The energy barrier for solution molecules to disrupt this bonded interface is elevated, thereby reducing transport. In Figure 2b, fewer solution molecules are adsorbed at higher Ca/Si ratios, diminishing the formation of a solution “barrier layer” near the interface. Consequently, the competitive effect of solution adsorption on CSH–γ–FeOOH bonding is suppressed, further favoring the direct CSH–γ–FeOOH interface. In Figure 2c, the near-constant *E*_γ–FeOOH/solutions_ values across Ca/Si ratios confirm that changes in transport properties are primarily governed by CSH’s structural evolution rather than γ–FeOOH’s affinity for the solution.

### 3.2. The Influence of Water Molecules on the Interfacial Adhesion Performance

The observed significant differences in interaction energy at γ–FeOOH/CSH interfaces with varying Ca/Si ratios under the same solution environment conditions warrant further mechanistic investigation. To elucidate the role of Ca/Si ratio in interfacial adhesion, CSH structures with different Ca/Si ratios were systematically analyzed through calculating the radial distribution function (RDF), coordination number (CN), and the number of hydrogen bond of Ca^2+^ ions and hydroxyl groups (O_h_ and H_o_) in the exposed area of the CSH surface with water molecules in solution, and analyzing the bonding interactions of each component.

**Figure 3:**
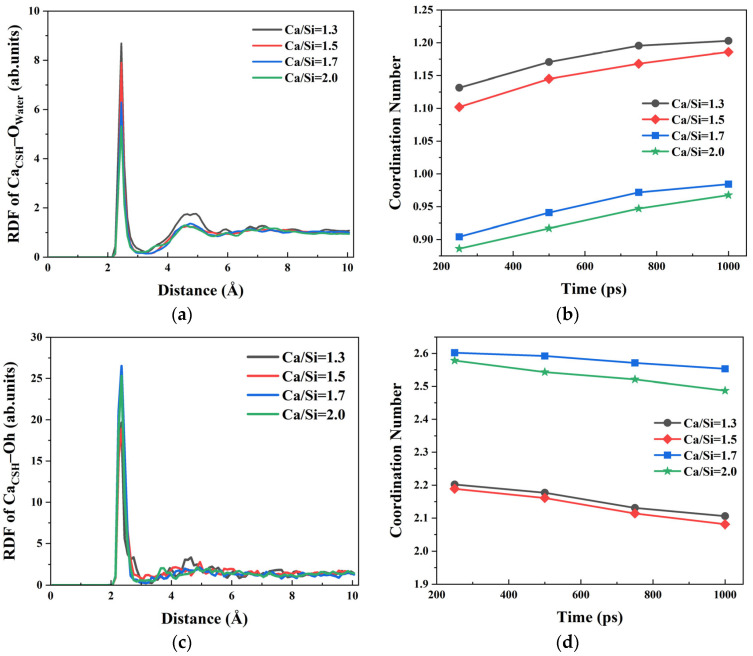
The RDF and CN between Ca^2+^ ions on the CSH surface and O atoms. (**a**) RDF of Ca_CSH_–O_water_; (**b**) CN of Ca_CSH_–O_water_; (**c**) RDF of Ca_CSH_–O_h_; (**d**) CN of Ca_CSH_–O_h_.

Firstly, the RDF and CN of Ca^2+^ ions on the surface of CSH among the oxygen atoms (O_Water_) in water molecules, the hydroxyl oxygen atoms in CSH, and oxygen atoms (O_h_ and O_s_) in silicon chains were calculated, respectively. The bonding differences of Ca^2+^ ions in the free regions of CSH structure surface under different Ca/Si ratios were characterized. The RDF curves between Ca_CSH_ and O_water_ under four different Ca/Si ratios revealed distinct bonding patterns, as shown in Figure 3a. A primary peak appeared at 2.45 Å in RDF curve, indicating that free Ca^2+^ ions on the CSH surface had a strong attraction effect on water molecules in the solution [44]. RDF peak intensities of Ca_CSH_–O_water_ decreased gradually from 8.688, 7.903, 6.288 to 5.315 as the Ca/Si ratio increased from 1.3, 1.5, 1.7 to 2.0. It denoted that the adsorption of Ca^2+^ ions on water molecules was weakened at higher Ca/Si ratios, consistent with the variation trend of *E*_CSH/solutions_ mentioned in Section 3.1. In addition, a secondary peak appeared at 4.55–4.95 Å near the primary peak of RDF curve, which demonstrates preserved long-range spatial correlations between Ca^2+^ ions and O_water_ regardless of Ca/Si ratio variations.

To quantitatively characterize the Ca_CSH_–O_water_ ionic bonding under varying Ca/Si ratios, the corresponding coordination numbers (CN) based on the RDF results were calculated (Figure 3b). The temporal evolution of CN values reveals the following distinct phases. When *t* = 0~800 ps, the CN value of Ca_CSH_–O_water_ ion pairs exhibited a monotonic increase, indicating hydration of surface Ca^2+^ ions progressively occurred. During the simulation process, the free Ca^2+^ ions on the surface of CSH gradually absorbed water molecules and form Ca_CSH_–O_water_ ionic bonds with water. When *t* > 800 ps, the increase rate of the Ca_CSH_–O_water_ CN value slowed down, reflecting the equilibrium state of the combination of CSH and water molecules in the solution. The four types of CSH with different Ca/Si ratios were compared. With the increase in the Ca/Si ratio, the CN value of Ca_CSH_–O_water_ gradually decreased, indicating that the surface free Ca^2+^ ions of the CSH structure with a high Ca/Si ratio had a weaker attraction to water, and the most pronounced reduction occurred when Ca/Si exceeded 1.5.

Although the variation trend of Ca_CSH_–O_water_ exhibits a certain regularity, analysis of the original structural models of CSH with different Ca/Si ratios reveals that the higher Ca/Si ratios correspond to more hydroxyl groups in the structure. Therefore, in addition to bonding with oxygen atoms in the solution, the interaction between free Ca^2+^ ions on CSH surface and hydroxyl oxygen atoms (O_h_) within CSH warrants investigation. The RDF curve of Ca_CSH_–O_h_ was calculated; results are presented in Figure 3c. The primary peak for CSH appeared at 2.35 Å with different Ca/Si ratios and was more pronounced than the corresponding RDF peak of Ca_CSH_–O_water_. This indicates that Ca^2+^ ions preferentially form Ca_CSH_–O_h_ ionic bonds with O_h_ atoms rather than Ca_CSH_–O_water_ bonds with water molecules. The CN values of Ca_CSH_–O_h_ are shown in Figure 3d. CSH with a higher Ca/Si ratio formed more Ca_CSH_–O_h_ ion pairs. During the simulation, as water molecules accumulated towards the CSH surface, the number of Ca_CSH_–O_h_ ion pairs decreased slightly. When the Ca/Si ratio was the same, the CN values of Ca_CSH_–O_h_ consistently exceeded those of Ca_CSH_–O_water_, indicating that Ca^2+^ ions were predominantly coordinated by O_h_ rather than O_water_. This preference suggests stronger interactions between Ca^2+^ ions and hydroxyl groups compared to water molecules in the CSH system.

**Figure 4:**
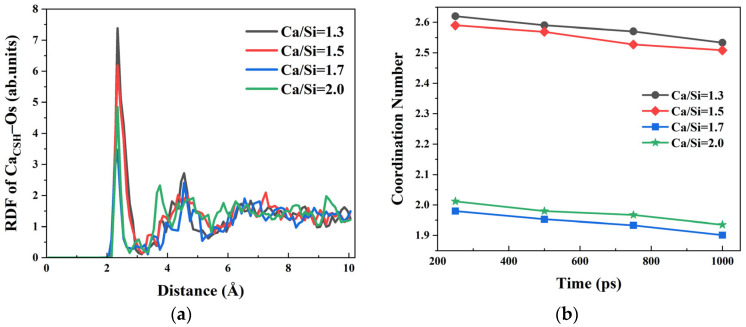
The RDF and CN between Ca^2+^ ions on the CSH surface and O_s_: (**a**) RDF of Ca_CSH_–O_s_; (**b**) CN of Ca_CSH_–O_s_.

Furthermore, as the Ca/Si ratio of CSH decreased, the number of silicon oxygen tetrahedra in the structure increased, enhancing the likelihood of Ca^2+^ ions bonding with bridging oxygen atoms (O_s_) in the tetrahedra, thereby impeding the formation of Ca_CSH_–O_water_ ion pairs. The RDF curves of Ca_CSH_–O_s_ shown in Figure 4a exhibit a consistent peak at 2.35 Å across all Ca/Si ratios, indicating a stable bonding distance between Ca^2+^ and O_s_ atoms. Notably, lower Ca/Si ratios yield higher RDF peak intensities, denoting that Ca^2+^ preferentially coordinates with O_s_ in CSH structures with more silicon oxygen tetrahedra. The CN numbers of the Ca_CSH_–O_s_ analysis at different simulation time points in Figure 4b reveal a parallel trend between Ca_CSH_–O_s_ and Ca_CSH_–O_h_, suggesting that the accumulation of water molecules on the CSH surface not only competes with O_h_ but also partially occupies the coordination sites of Ca_CSH_–O_s_ ion pairs.

Based on the results mentioned above, the relative strength of ionic bonding between Ca^2+^ ions and the three oxygen species follows the order: Ca_CSH_–O_h_ > Ca_CSH_–O_s_ > Ca_CSH_–O_water_. However, the variation trends of RDF peak intensities with Ca/Si ratio exhibit inconsistencies between Ca_CSH_–O_s_ and Ca_CSH_–O_h_ systems. Specifically, in the Ca_CSH_–O_h_ RDF profile, the peak intensity for Ca/Si = 2.0 is lower than that for Ca/Si = 1.7, while in the Ca_CSH_–O_s_ system, the peak intensity is 4.851 at Ca/Si = 2.0, which significantly exceeds the intensify of 3.466 at Ca/Si = 1.7. This apparent discrepancy primarily stems from distinct spatial configurations and distributions of silicon oxygen tetrahedra on CSH surfaces with different Ca/Si ratios. In the Ca/Si = 2.0 CSH structure, the silicon chain network adopts a more dispersed arrangement, which facilitates more migration of free Ca^2+^ ions into the structural interlayers, particularly into the first layer. Furthermore, Ca^2+^ ions in the first layer demonstrate preferential coordination with O_s_ atoms, while Ca^2+^ ions in the second layer exhibit stronger affinity for O_h_ groups.

By analyzing the bonding interactions between Ca^2+^ ions in the free zone of CSH surface and oxygen atoms in other components, it was found that the crystal structure of CSH with varying Ca/Si ratios influenced the distribution of Ca^2+^ ions. As a result, it subsequently altered the hydration behavior of free Ca^2+^ ions on the CSH surface and ultimately led to differences in the adhesion performance between CSH and γ–FeOOH.

The results demonstrate that free Ca^2+^ ions on the surface of CSH with high Ca/Si ratio form fewer bonds with water molecules in the solution. Ca^2+^ ions preferentially interact with the free hydroxyl groups on the surface of CSH to form Ca_CSH_–O_h_ bonds. During the simulation progress, the free Ca_CSH_–O_h_ bonds exhibited higher mobility toward the surface of γ–FeOOH and formed O_h_–Ca_CSH_–O_γ–FeOOH_ with it, thereby effectively reducing the inhibitory effect of water molecules on the interfacial interaction between CSH and γ–FeOOH.

**Figure 5:**
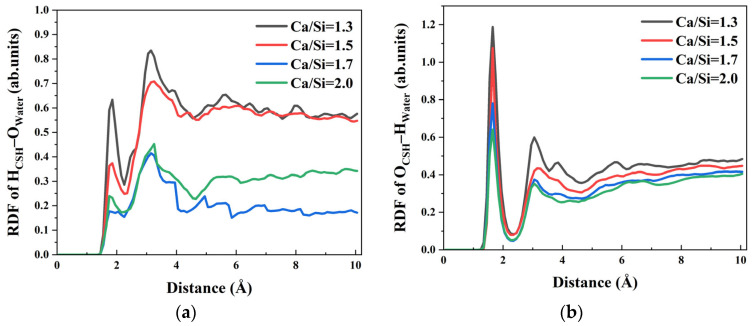
The RDF of H_CSH_–O_water_ andO_CSH_–H_water_: (**a**) RDF of H_CSH_–O_water_; (**b**) RDF of O_CSH_–H_water_.

In addition to the free Ca^2+^ ions, the exposed hydroxyl groups (H_CSH_ and O_CSH_) on the CSH surface with different Ca/Si ratios also formed chemical bonds with other components, thereby influencing the interfacial interaction between CSH and γ–FeOOH. The RDF curves between surface hydroxyl groups of CSH with different Ca/Si ratios and water molecules in the solution are shown in Figure 5. As illustrated in Figure 5a, the first peak of of H_CSH_–O_water_ in RDF appeared at 1.95 Å, followed by a second peak at 3.15 Å. Meanwhile, Figure 5b shows that O_CSH_–H_water_ in RDF had its first peak at 1.65 Å and a second peak at 3.05 Å. The positions of the first peaks of both H_CSH_–O_water_ and O_CSH_–H_water_, the most probable distance between the two atoms, are shorter than the typical hydrogen bond length (2.45 Å), indicating that CSH can act as both a hydrogen bond donor and acceptor with water molecules in solutions of varying Ca/Si ratios.

Comparing CSH with different Ca/Si ratios, the peak intensities of H_CSH_–O_water_ and O_CSH_–H_water_ in RDF curves gradually increase with the decrease in Ca/Si ratio, suggesting stronger hydrogen bonding interactions between CSH with low–Ca/Si ratios and water molecules. For example, in O_CSH_–H_water_ RDF curves, as the Ca/Si ratio increases from 1.3, 1.5, 1.7 to 2.0, the peak values decrease from 1.187, 1.075, 0.781 to 0.641, respectively. This trend demonstrates that a lower Ca/Si ratio enhances the hydrogen bond strength between CSH and water molecules.

Since the H_CSH_ and O_CSH_ groups on CSH surface preferentially form hydrogen bonds with O_water_ and H_water_ (H_CSH_–O_water_ and O_CSH_–H_water_), the coordination sites that could originally bind to hydroxyl groups on the γ–FeOOH surface are occupied by water molecules. Consequently, the number and strength of hydrogen bonds (H_CSH_–O_γ–FeOOH_ and O_CSH_–H_γ–FeOOH_) formed between CSH and γ–FeOOH are significantly reduced.

In summary, CSH with varying Ca/Si ratios forms Ca_CSH_–O_water_ ionic bonds and hydrogen bonds (H_CSH_–O_water_ and O_CSH_–H_water_) with water molecules in solution. These bonding interactions occupy the coordination sites on the CSH surface, thereby weakening the interfacial interaction between γ–FeOOH and CSH. The comparative analysis of the RDF peak values for Ca_CSH_–O_water_, H_CSH_–O_water_, and O_CSH_–H_water_ under different Ca/Si ratios reveals an increasing trend in peak intensity with decreasing Ca/Si ratio. This phenomenon indicates that CSH with lower Ca/Si ratios exhibits stronger binding with water molecules, which more effectively inhibits the interfacial interaction between γ–FeOOH and CSH. Consequently, this competitive bonding mechanism ultimately leads to the deterioration of bonding performance at the γ–FeOOH/CSH interface.

### 3.3. The Influence of Erosive Ions on the Interfacial Adhesion Performance

In the molecular dynamics simulation of γ–FeOOH/CSH interfacial bonding under varying Ca/Si ratios, NaCl + Na_2_SO_4_ composite solution was employed to simulate the external corrosive environment. Beyond examining the interaction between water molecules and CSH with four distinct Ca/Si ratios, the effects of Na^+^, Cl^−^, and SO_4_^2−^ ions on interfacial bonding performance were systematically investigated. This section elucidates the degradation mechanisms induced by each ionic from a microscopic perspective.

**Figure 6:**
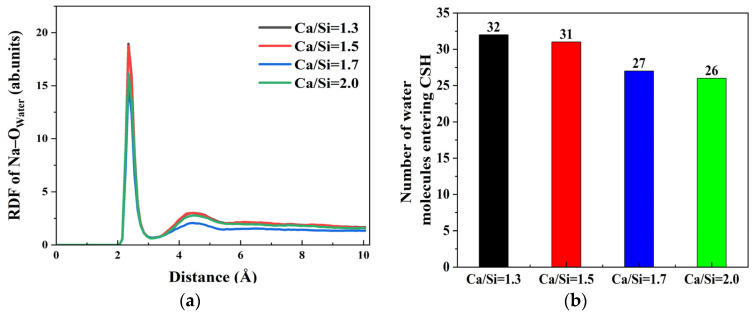
The attraction mechanism of Na^+^ ions to water molecules: (**a**) RDF of Na–O_water_; (**b**) number of water molecules entering CSH.

Notably, Na^+^ ions tend to accumulate on the CSH surface, promoting water molecule ingress into the CSH matrix. However, the adsorption characteristics under varying CSH crystal structures induced by different Ca/Si ratios require further exploration. Through the RDF results of Na–O_water_ at γ–FeOOH/CSH interfaces with different Ca/Si ratios, as shown in Figure 6a, the influence of Na^+^ ions on the interaction of γ–FeOOH/CSH interface with different Ca/Si ratios was evaluated. The RDF profiles exhibit a pronounced peak at 2.35 Å across all four Ca/Si ratios, indicating persistent strong Na^+^–water interactions regardless of CSH composition. This peak corresponds to a well-defined hydration shell where water molecules coordinate Na^+^ ions at this characteristic distance. Moreover, the peak intensity demonstrates an inverse relationship with Ca/Si ratio, revealing that higher Ca/Si ratios in CSH attenuate the secondary hydration effects of Na^+^ ions in solution.

This trend is further corroborated by Figure 6b, demonstrating an inverse correlation between Ca/Si ratio and water molecule penetration into the CSH surface. Quantitative analysis reveals that at Ca/Si = 2.0, approximately 26 water molecules access the CSH surface, while this number increases to 32 when Ca/Si decreases to 1.3. The findings prove that Ca/Si ratio modulates the hydration capability of Na^+^ ions. Higher Ca/Si ratios attenuate the hydration strength of surface Na^+^ ions, consequently reducing the possibility of water molecules bonding with CSH. The phenomenon promotes the migration of free Ca^2+^ ions and hydroxyl groups (O_h_ and H_o_) from CSH to γ–FeOOH surfaces for interfacial bonding. Ultimately, CSH with elevated Ca/Si ratios exhibits weaker solution interactions but stronger interfacial bonding with γ–FeOOH.

**Figure 7:**
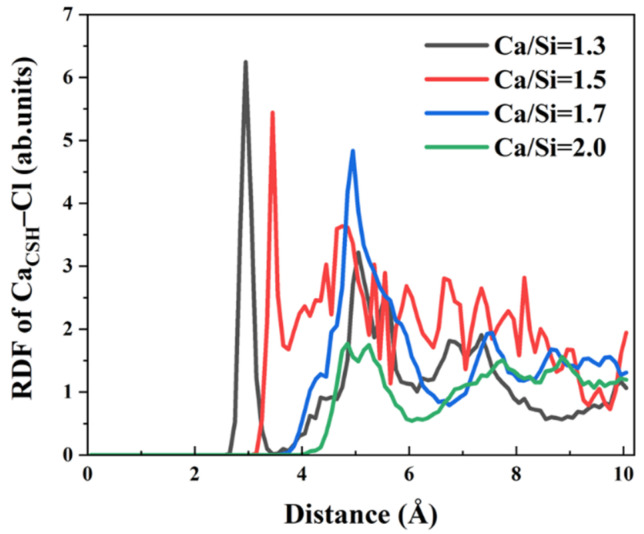
RDF of Ca_CSH_–Cl.

In addition to Na^+^ ions, the interfacial behavior of Cl^−^ and SO_4_^2−^ anions at varying Ca/Si ratios warrants systematic investigation. Owing to electrostatic attraction, Cl^−^ ions form Ca_CSH_–Cl ionic bonds with Ca^2+^ ions in the free zone of CSH surface. To elucidate the effect of Ca/Si ratios on these interactions, RDF curves of Ca_CSH_–Cl were calculated across four Ca/Si ratios in Figure 7. The results reveal significant Ca/Si–dependent variations in bond characteristics. When Ca/Si = 1.3, 1.5, and 1.7, the peak position of RDF gradually increases from 2.95 Å, 3.45 Å to 4.95 Å. When Ca/Si = 2.0, no distinct peak appears. The peak position represents the distance of interaction between Ca^2+^ ions on the CSH surface and Cl^−^ ions in the solution. Therefore, when Ca/Si = 1.3, Cl^−^ ions aggregate at a distance of 2.95 Å around Ca^2+^ ions. As the Ca/Si ratio increases, Cl^−^ ions gradually aggregate at farther positions on the CSH surface until there is no significant aggregation. Peak intensity attenuates with increasing Ca/Si ratio, indicating that the bonding quantity and strength of the two ions gradually weaken. It can be concluded that lower Ca/Si ratios promote Cl^−^ coordinate to surface Ca^2+^ ions, while higher ratios progressively weaken this interaction. Consequently, CSH surfaces with high Ca/Si ratio exhibit reduced Cl^−^ binding probability, liberating more Ca^2+^ ions for γ–FeOOH interfacial bonding.

**Figure 8:**
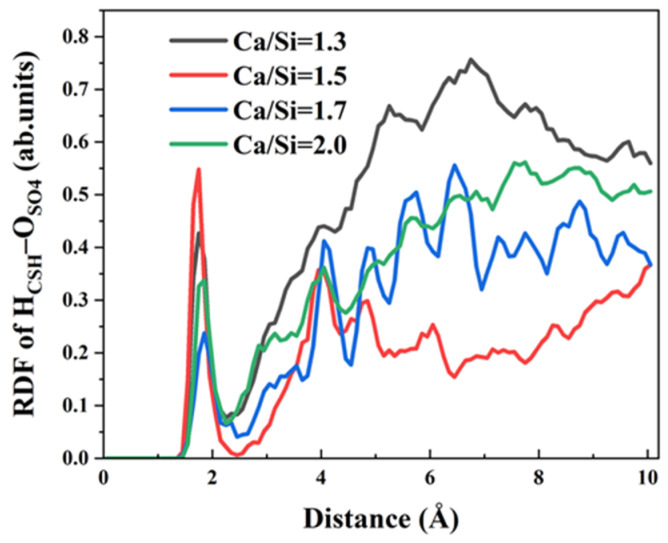
RDF of H_CSH_–O_SO4_.

The chemical bonding mechanisms differ between Cl^−^ and SO_4_^2−^ ions. Each SO_4_^2−^ anion possesses four oxygen atoms, providing four potential coordination sites for hydrogen bond formation with CSH. To elucidate the influence of varying Ca/Si ratios on the hydrogen bonds formation between SO_4_^2−^ and CSH, RDFs between O_SO4_ and H_CSH_ were analyzed, as presented in Figure 8. The key observations include the following points. Firstly, all Ca/Si ratios exhibit a distinct H_CSH_–O_SO4_ RDF peak at 1.85 Å, well within the typical hydrogen bonding distance threshold of 2.45 Å, confirming strong hydrogen bonding interactions. Secondly, comparative analysis reveals significantly higher peak intensities at lower Ca/Si ratios (1.3 and 1.5) compared to higher ratios (1.7 and 2.0), demonstrating that high Ca/Si ratios suppress H_CSH_–O_SO4_ hydrogen bond formation. It is hard for hydrogen atoms on a CSH surface with a high Ca/Si ratio to bind to SO_4_^2−^ ions in solution and form hydrogen bonds. Thirdly, the suppression effect above liberates more H_CSH_ sites for interfacial bonding with γ–FeOOH, facilitating the formation of stronger H_CSH_–O_γ–FeOOH_ hydrogen bonds that enhance overall CSH/γ–FeOOH interaction energy.

### 3.4. Transport Behaviour of Solutions in Pores with Varying Ca/Si Ratios

**Figure 9:**
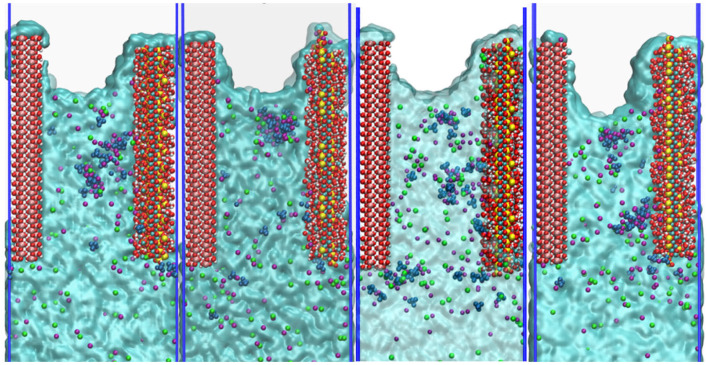
Snapshot of transport of solution in pores with different Ca/Si at 2000 ps.

While the interfacial bonding behavior between CSH and γ–FeOOH elucidates the structural characteristics, the ion transport within the nanopore further reflects its dynamic functional performance. Figure 9 presents capillary transport snapshots of NaCl + Na_2_SO_4_ solution in CSH/γ–FeOOH nanopores with varying Ca/Si ratios of 1.3, 1.5, 1.7, and 2.0 when the simulation time *t* = 2000 ps [45]. From the aspect of the water transport dynamics, it is observed that complete surface coverage by water molecules occurs across all Ca/Si ratios, which indicates that strong hydrophilic attraction from CSH substrates with varying Ca/Si ratios drives water molecules’ migration. However, the specific impact of CSH with varying Ca/Si ratios on the transport velocity of water molecules still requires further investigation. Compared with water molecules, the movement velocity of Na^+^, Cl^−^, and SO_4_^2−^ ions is much slower. This indicates that ion transport is partially dependent on water molecular driving force to move forward. Differences in ion invasion depths are also apparent. When CSH Ca/Si ratio = 1.3, 1.5, and 1.7, they occupy about 75% of the entire pore length. When CSH Ca/Si ratio increases to 2.0, the ion invasion depth decreases to about 70% of the entire pore. In terms of the interfacial distribution, ions showed pronounced ion accumulation preference for CSH over γ–FeOOH interfaces. Particularly, SO_4_^2−^ exhibits significant clustering behavior, forming multiple large ion clusters and adsorbing on the CSH surface. The phenomena above indicate that the transport mechanism not only involves the diffusion of the solution itself, but is also affected by the matrix interface interaction, and the interaction with CSH is stronger than that with γ–FeOOH.

The preceding analysis demonstrates that variations in CSH crystal structure induced by different Ca/Si ratios influence the transport behavior of NaCl + Na_2_SO_4_ composite solution within the nanopores. To quantitatively characterize the transport differences, the temporal evolution of penetration depths for water molecules and ions (Na^+^, Cl^−^, and SO_4_^2−^) along the pore surfaces was systematically recorded. Considering the invariant structure of γ–FeOOH substrates and their negligible ion adsorption capacity, the capillary transport behavior was quantified by analyzing ion penetration profiles along CSH surfaces with varying Ca/Si ratios.

**Figure 10:**
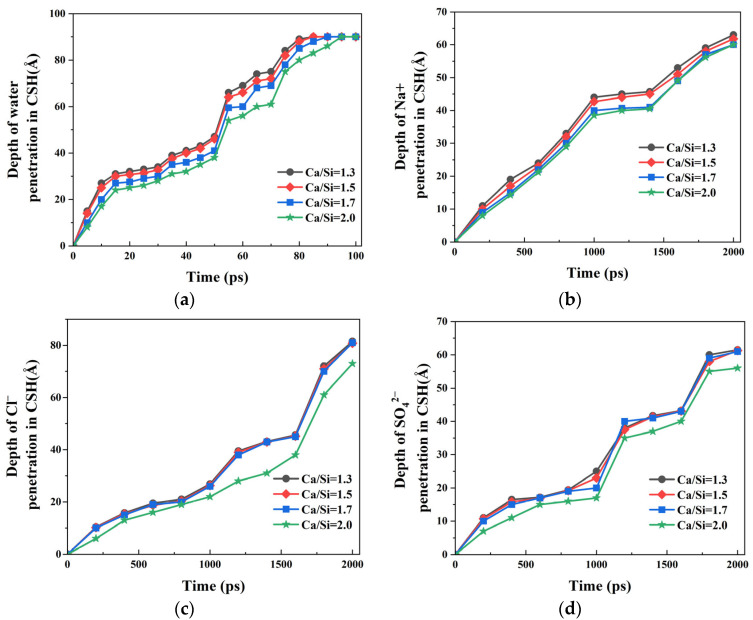
Depth of invasion along the surface of CSH: (**a**) water; (**b**) Na^+^; (**c**) Cl^−^; (**d**) SO_4_^2−^.

The penetration depth profiles of water molecules on CSH surfaces with different Ca/Si ratios is presented in Figure 10a. Complete surface coverage was achieved by *t* = 100 ps in all cases. It was observed that the higher the Ca/Si ratio, the smaller the penetration depth of water molecules on the CSH surface. When *t* = 75 ps, the measured penetration depths were 84 Å, 82 Å, 78 Å, and 75 Å for CSH Ca/Si ratios of 1.3, 1.5, 1.7, and 2.0, respectively. The decrease in the penetration depth with the increase in the Ca/Si ratio indicates that in systems with a high Ca/Si ratio, the interaction at the water–CSH interface weakens, resulting in the slow transport of water molecules.

Figure 10b–d illustrate the penetration depths of Na^+^, Cl^−^, and SO_4_^2−^ ions on CSH surfaces with varying Ca/Si ratios. It can be concluded that all ions exhibit significantly shallower penetration than water molecules at all timepoints, confirming preserved transport asynchronicity regardless of Ca/Si ratio variations. The ion penetration depths on the CSH surface keep decreasing as Ca/Si ratios increases. Compared with Na^+^ ions, Cl^−^ and SO_4_^2−^ show greater Ca/Si ratio dependence, particularly when Ca/Si = 2.0 where divergence was most significant. Quantitative analysis of the data at *t* = 1600 ps reveals that when *t* = 1600 ps, the penetration depth of Na^+^ ions on CSH interface with Ca/Si = 1.3 was measured to be 53 Å. When the Ca/Si ratio increased to 2.0, the penetration depth decreased by 7.54%. Under identical conditions, Cl^−^ ions exhibited a more substantial reduction in penetration depth by 16.66%. These results demonstrate that increasing the Ca/Si ratio of CSH induces selective ion filtration, with enhanced filtration efficiency observed for Cl^−^ and SO_4_^2−^ ions compared to Na^+^ ions at Ca/Si = 2.0. The differential filtration effects suggest that the interfacial interactions are strongly influenced by both ionic charge and hydration characteristics [46,47].

The results presented in Figure 10c demonstrate that the penetration rate of Cl^−^ ions is significantly reduced in CSH nanochannels with higher Ca/Si ratios, indicating an accumulation and prolonged retention of chloride ions within the nanopore. This observation is in good agreement with the experimental findings reported by Zhou et al. [22]. Through systematic experimental investigations, Zhou examined the transport and adsorption behaviors of water molecules, Ca^2+^ ions, and Cl^−^ ions in CSH nanochannels with varying Ca/Si ratios. Their immersion tests clearly showed that CSH specimens with higher Ca/Si ratios exhibit enhanced chloride ion adsorption capacity.

The NaCl + Na_2_SO_4_ composite solution exhibited significantly different transport behaviors in the γ–FeOOH/CSH nanopores with four different Ca/Si ratios. This correlation suggests that structural modifications in CSH directly influence both the quantity and strength of interfacial bonds formed with water molecules and ions. The results showed that systems with higher Ca/Si ratios demonstrated significantly reduced transport rates for both aqueous and ionic components. Although these observations clearly establish the Ca/Si ratio as a critical regulator of transport phenomena, the precise molecular-scale bonding mechanisms responsible for the blocking effect require further investigation.

### 3.5. Differences in Bonding Between Solutions and CSH with Different Ca/Si

**Figure 11:**
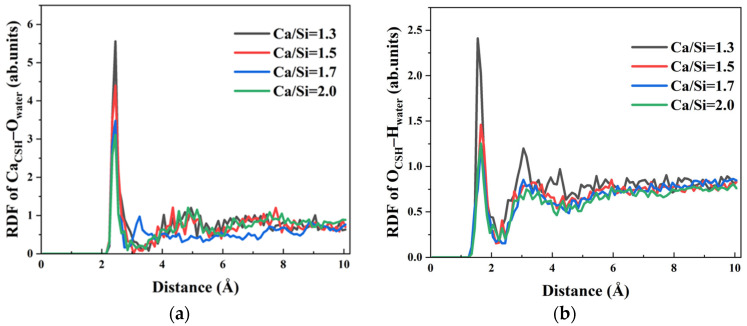
RDF curve of Ca_CSH_–O_water_ and O_CSH_–H_water_: (**a**) RDF of Ca_CSH_–O_water_; (**b**) RDF of O_CSH_–H_water_.

In order to elucidate the influence mechanism of CSH with different Ca/Si ratios on solution transport behaviour, the RDFs between Ca^2+^ ions and hydroxyl groups (O_h_ and H_o_) in the exposed regions of CSH surface and the surrounding solution were assessed. Initial investigation focused on water–CSH interactions across different Ca/Si ratios. The RDF curves of O_water_–Ca_CSH_ under four different Ca/Si ratios were shown in Figure 11a, revealing a characteristic first peak at 2.45 Å. The peak position confirms strong chemical bonding between O_water_ and Ca^2+^ ions on CSH surfaces with varying Ca/Si. This bonding is also one of the primary driving forces for promoting the forward transport of water molecules within γ–FeOOH/CSH pores.

The peak intensity showed a progressive decrease from 5.558, 4.401, 3.478 to 3.10 as the Ca/Si ratio increased from 1.3 to 2.0. This indicates that the CSH structure with higher Ca/Si ratio attenuated both the quantity and strength of chemical bonds between Ca^2+^ ions on the surface and water molecules, consequently weakening the water–CSH interfacial attraction. The observed RDF variations originate fundamentally from structural differences in the initial CSH models. Specifically, CSH with higher Ca/Si ratios contains more hydroxyl groups within its molecular structure. The hydroxyl oxygen atoms (O_h_) in CSH preferentially occupy coordination sites of Ca^2+^ ions, forming stable Ca_CSH_–O_h_ ion pairs. When water molecules in the solution approach the CSH surface, the number of free Ca^2+^ ions that can bond with O_water_ on the surface is reduced, thereby decreasing Ca_CSH_–O_water_ interactions.

Hydroxyl groups on CSH surfaces additionally participate in hydrogen bonding with water molecules, constituting another significant driving force for aqueous transport. The RDF curves of O_CSH_–H_water_ under varying Ca/Si ratios were calculated, as shown in Figure 11b. The first peak positions of the curves are distributed in the range of 1.55 Å~1.65 Å, which is shorter than the characteristic hydrogen bond distance of 2.45 Å. This indicates that CSH forms hydrogen bonds with water molecules in solution under all Ca/Si ratios. The Ca/Si ratio of CSH increases with the gradual RDF peak reduction in O_CSH_–H_water_, indicating a progressive weakening of hydrogen bond strength with increasing Ca/Si ratio. Particularly, when Ca/Si = 1.3, it exhibits much higher hydrogen bonding capacity compared to high Ca/Si ratio structure. Specifically speaking, peak intensity attenuates by 39.87%, 48.15%, and 49.73% with Ca/Si ratio increases from 1.3, 1.5, 1.7 to 2.0, respectively.

The analysis demonstrates that CSH with varying Ca/Si ratios facilitates water molecule transport through formation of Ca_CSH_–O_water_ and O_CSH_–H_water_ bonds, enabling complete substrate surface coverage by water. However, increasing Ca/Si ratio modifies the CSH crystal structure, leading to the strength weakening of Ca_CSH_–O_water_ and O_CSH_–H_water_ bonds formed on surface free regions. This attenuation reduces the driving force for water molecule transport, ultimately resulting in decreased penetration depths observed for high Ca/Si ratio CSH surfaces at equivalent timepoints.

**Figure 12:**
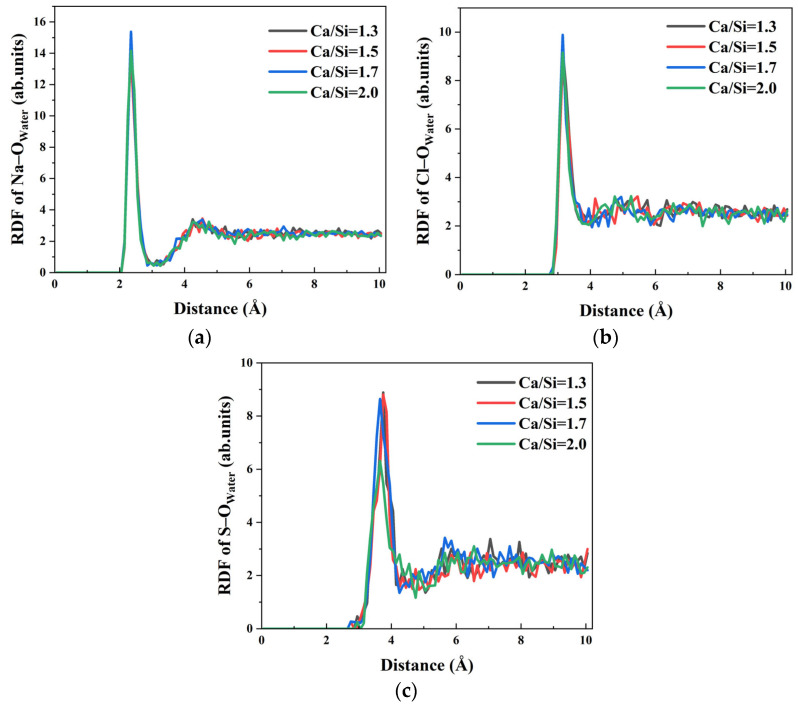
RDF of water molecules and ions in solution: (**a**) RDF of Na–O_water_; (**b**) RDF of Cl–O_water_. (**c**) RDF of S–O_water_.

Ion transport within the capillary system derives partially from the driving effect of water molecules. RDF analysis of Na–O_water_, Cl–O_water_, and S–O_water_ interactions in Figure 12 reveals that the first peaks appear at 2.35 Å, 3.15 Å, and 3.65 Å, respectively, confirming strong hydration effects for all ions across different Ca/Si ratios, while Na–O_water_ and Cl–O_water_ interactions show minimal Ca/Si ratio dependence, S–O_water_ bonding exhibits significant sensitivity. Specifically, the hydration of SO_4_^2−^ remains consistent when Ca/Si ratios = 1.3, 1.5, and 1.7, but shows marked weakening at Ca/Si = 2.0 as evidenced by reduced RDF peak intensity. The decreased S–O_water_ interaction directly correlates with the observed block of SO_4_^2−^ transport in systems with high Ca/Si ratio.

In addition to the water–ion bonding cooperation that facilitates ions transport through the pores, ion adsorption by the matrix on both interfaces of the pores promotes the continuous transport of ions. Given the invariant γ–FeOOH crystal structure with all Ca/Si ratio models and Na^+^, Cl^−^, and SO_4_^2−^ ions, ions tend to accumulate on CSH surfaces. As demonstrated in Section 3.4, the difference in ion adsorption primarily stems from variations in CSH–ion bonding characteristics.

**Figure 13:**
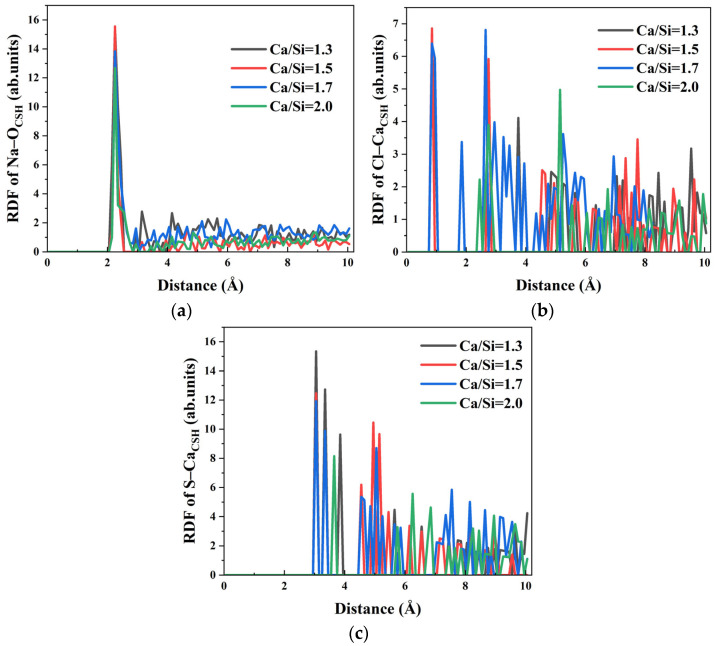
RDF between ions and CSH: (**a**) RDF of Na–O_CSH_; (**b**) RDF of Cl–Ca_CSH_; (**c**) RDF of S–Ca_CSH_.

RDF analysis of Na–O_CSH_, Cl–O_CSH_, and S–O_CSH_ interactions in Figure 13 reveals continuous attenuation of peak intensities with increasing Ca/Si ratios. This trend indicates progressively weaker bonding between various ions in the solution and the available surface sites (free hydroxyl groups and Ca^2+^ ions) on CSH with high Ca/Si ratio. Notably, while the first peak in Na–O_CSH_ RDF remains constant at 2.25 Å at all Ca/Si ratios, both Cl–O_CSH_ and S–O_CSH_ exhibit significant Ca/Si–dependent peak shifts. Specifically, when Ca/Si ratios = 1.3, 1.5 and 1.7, the first peaks of Cl–O_CSH_ and S–O_CSH_ appear at 0.85 Å and 3.05 Å, respectively. When Ca/Si ratio = 2.0, the peak positions shift outward to 2.45 Å and 3.65 Å. The substantial increase in equilibrium bonding distances, coupled with reduced peak intensities, confirms weakened Cl–O_CSH_ and S–O_CSH_ interactions at high Ca/Si ratios. The phenomenon correlates with the penetration depth results in Figure 10, explaining the enhanced filtration effect of Cl^−^ and SO_4_^2−^ ions observed at Ca/Si = 2.0 through weakened interfacial bondings.

## 4. Conclusions

This study employed molecular dynamics simulations to investigate the adhesion and transport characteristics of γ–FeOOH/CSH interfaces formed by combining four distinct CSH crystal models (Ca/Si = 1.3, 1.5, 1.7, and 2.0) with γ–FeOOH in a NaCl + Na_2_SO_4_ composite solution environment. The following conclusions can be drawn from the calculation and analysis of interaction energy, RDFs, coordination numbers, hydrogen bonding, and penetration depths:

1. The change in Ca/Si ratio had a significant impact on the interaction energy between CSH and γ–FeOOH, as well as between CSH and solution. As the Ca/Si ratio increased from 1.3 to 2.0, the CSH/γ–FeOOH interaction energy increased by 48.7%, while CSH/solution interaction energy decreased by 10.63%. Optimal interfacial bonding between CSH and γ–FeOOH occurred at Ca/Si = 2.0;
2. The bonding between water in solution and CSH with high Ca/Si ratios is attenuated, leading to the reduced coordination site occupancy by water. The formation possibility of hydrogen bonds such as Ca_CSH_–O_water_, H_CSH_–O_water_, and O_CSH_–H_water_ bonds is decreased, thereby enhancing CSH preferential bonding with γ–FeOOH to form Ca_CSH_–O_γ–FeOOH_, H_CSH_–O_γ–FeOOH_, and O_CSH_–H_γ–FeOOH_ hydrogen bonds. As a result, the interaction between γ–FeOOH and CSH is elevated;
3. CSH with high Ca/Si ratios modifies ionic effects in the following ways. Inhibiting the secondary hydration of Na^+^ ions on water molecules reduces Na–O_water_ bond strength and weakens the influence of water molecule degradation on the interfacial adhesion performance. Reducing the interfacial bonding performance of Cl^−^ and SO_4_^2−^ ions has an affect on the free Ca^2+^ ions and hydrogen atoms on the CSH surface. The decreased bonding effects of Ca_CSH_–Cl and H_CSH_–O_SO4_ bonds weakens interfacial bonding performance.
4. The transport characteristics in γ–FeOOH/CSH nanopores with high Ca/Si ratio include the overall retardation of water and ion transport rates and significant variations in penetration depths. Compared with Na^+^ ions, the filtration selectivity on Cl^−^ ions and SO_4_^2−^ ions is enhanced when the Ca/Si ratio of CSH increases to 2.0;
5. The higher the Ca/Si ratio of CSH, the lower the strength and quantity of Ca_CSH_–O_water_ and O_CSH_–H_water_ bonds, which weakens the adsorption of water molecules by the matrix and slows down the invasion rate of water molecules. The decreasing transport rate of Na^+^ and Cl^−^ ions in γ–FeOOH/CSH nanopores with high Ca/Si ratio results from the weakened interaction between Na–O_CSH_ and Cl–Ca_CSH_. The decreasing transport rate of SO_4_^2−^ ions is influenced by both S–Ca_CSH_ ion pairs and S–O_water_ bonds. The interaction between Cl–Ca_CSH_, S–Ca_CSH_, and S–O_water_ ion pairs is weakened the most when Ca/Si = 2.0.

The findings of this study suggest that increasing the Ca/Si ratio of cementitious materials can enhance their resistance to marine erosion. For instance, in concrete preparation, using high-calcium Portland cement or low-silica quartz sand can improve the durability of marine structures by strengthening the interfacial bonding between CSH and γ–FeOOH and reducing the penetration of corrosive ions. While this work primarily focuses on the mechanical, wetting, and transport properties of the CSH matrix, further investigations should address the interfacial adhesion and ion transport behavior between steel fibers and CSH. Additionally, comprehensive simulations are needed to explore the performance of concrete reinforced with steel fibers of varying compositions under aggressive environments.

## Figures and Tables

**Table 1:** Q species percentage distribution [20].

	Q^0^ (%)	Q^1^ (%)	Q^2^ (%)
Ca/Si = 1.3	0	43.24	56.76
Ca/Si = 1.5	0	56.25	43.75
Ca/Si = 1.7	1.22	80.49	18.29
Ca/Si = 2.0	8.45	78.87	12.68

## Data Availability

The original contributions presented in this study are included in the article. Further inquiries can be directed to the corresponding author.
